# Labeling Stem Cells with a New Hybrid Bismuth/Carbon Nanotube Contrast Agent for X-Ray Imaging

**DOI:** 10.1155/2019/2183051

**Published:** 2019-06-11

**Authors:** Mayra Hernández-Rivera, Stephen Y. Cho, Sakineh E. Moghaddam, Benjamin Y. Cheong, Maria da Graça Cabreira-Hansen, James T. Willerson, Emerson C. Perin, Lon J. Wilson

**Affiliations:** ^1^Department of Chemistry MS-60, Rice University, P.O. Box 1892, Houston, TX 77251, USA; ^2^CHI St. Luke's Health—Baylor St. Luke's Medical Center, 6720 Bertner Ave., MC 2-270, Houston, TX 77030, USA; ^3^Texas Heart Institute, 6770 Bertner Ave C350, Houston, TX 77030, USA

## Abstract

The poor retention and survival of cells after transplantation to solid tissue represent a major obstacle for the effectiveness of stem cell-based therapies. The ability to track stem cells *in vivo* can lead to a better understanding of the biodistribution of transplanted cells, in addition to improving the analysis of stem cell therapies' outcomes. Here, we described the use of a carbon nanotube-based contrast agent (CA) for X-ray computed tomography (CT) imaging as an intracellular CA to label bone marrow-derived mesenchymal stem cells (MSCs). Porcine MSCs were labeled without observed cytotoxicity. The CA consists of a hybrid material containing ultra-short single-walled carbon nanotubes (20–80 nm in length, US-tubes) and Bi(III) oxo-salicylate clusters which contain four Bi^3+^ ions per cluster (Bi_4_C). The CA is thus abbreviated as Bi_4_C@US-tubes.

## 1. Introduction

In recent years, stem cell research has taken many new directions. In particular, adult stem cells are being used as vectors to provide extracellular survival and growth factors in tissue repair, as well as, to treat disease by replacing damaged tissue [1]. Clinical trials involving cell-based autologous therapies (patients receive their own cells) and allogenic therapies (recipients receive cells from a donor) have been conducted for a plethora of diseases, such as diabetes, paraplegia, multiple sclerosis, critical limb ischemia, cerebrovascular diseases, blood-related cancers, and some solid tumors [1–3]. Among the adult stem cells found in the human body, multipotent mesenchymal stromal cells (MSCs) have been widely used due to their relatively easy isolation and ex vivo expansion. In addition, MSCs have the advantage of being less immunogenic than other stem cells due to the lack of costimulatory agents of the B7 family that are required to initiate an immune response [4–6]. This allows the use of MSCs without concerns about immunological rejection or the need for immunosuppressant drugs, making MSCs a universal stem cell source. With the rapid increase of reported cases of MSC-based therapies, there is an urgent need to track the cells *in vivo* during preclinical and clinical trials to further understand and evaluate the behavior and fate of transplanted MSCs.

To address this need, various imaging techniques have been proposed and investigated, including X-ray computed tomography (CT) imaging [7], magnetic resonance imaging (MRI) [8–13], optical imaging (such as bioluminescence and fluorescence) [14–16], ultrasound-guided photoacoustic (US/PA) imaging [17], single-photon emission computed tomography (SPECT) imaging [18], and positron emission tomography (PET) imaging [19]. Of these techniques, optical imaging has been shown to be a potent tool in preclinical small animal studies, but its use alone is not yet translatable to clinical practice due to its low tissue penetration depth [20, 21]. The use of each of these imaging techniques for stem cell tracking is reviewed elsewhere [20]. Currently, the most used and preferable imaging modality for stem cell tracking is MRI with *T*_2_/*T*_2_^*∗*^ contrast acquired when stem cells are labeled with superparamagnetic agents such as iron oxide nanoparticles [22–24]. However, a significant clinical limitation of MRI is its incompatibility with various medical and life support devices such as pacemakers and defibrillators whose presence can cause serious issues (i.e., magnetic field interactions, heating, and other artifacts). Another disadvantage is the long scan time (20–90 min), which requires patients to remain still in an enclosed space, potentially causing discomfort, anxiety, and claustrophobia. X-ray CT, although it is based on ionizing radiation, it provides a number of advantages; for example, it requires much less time per acquisition (each scan can be performed in less than 1 min) and does not possess a harm for patients with transplanted magnetics medical devices. Also, X-ray CT and other X-ray-based imaging modalities are often more readily available than other imaging technologies, especially in countries that are less industrialized and economically developed [25–27].

In a previous publication, we reported the use of CT in combination with a carbon nanotube- (CNT-) based contrast agent (CA) to track stem cells [7]. X-ray CAs (or radiocontrast agents) are used to provide transient contrast enhancement in X-ray-based imaging modalities such as radiography, CT, and fluoroscopy and are currently under investigation for cell labeling [28]. Contrast enhancement comes largely from the photoelectron effect due to high atomic numbers. As a rule, materials possessing a higher density (*ρ*) and high atomic number (*Z*) absorb X-rays more effectively [29, 30]. The capability of matter to attenuate X-rays is measured in Hounsfield units (HU). By definition, water has a HU value of 0, and air has a value of −1000 HU, while most soft tissues fall within 30–100 HU. The HU of a material with a linear X-ray absorption coefficient (*μ*) is defined as [29, 30]

(1) $$\begin{array}{c} \text{HU}=\frac{\left(\mu -\mu_{\text{water}}\right)}{\mu_{\text{water}}}\times 1000. \end{array}$$

Radiocontrast agents with good X-ray attenuation (high HU values) facilitate the process of distinguishing the region of interest by increasing the HU value of the tissue of interest relative to the background. Currently, there are two types of CAs approved for human use: barium sulfate suspensions (*Z*_Ba_ = 56) and small water-soluble iodinated molecules (*Z*_I_ = 53) [29]. However, both of these types of agents are exclusively for extracellular use. More recently, research has been focused on the use of small molecules or nanoparticles containing atoms with higher atomic numbers. For example, gold nanoparticles (*Z*_Au_ = 79) are an ideal alternative since gold has both a high density and a high atomic number, and it provides about 2.7 times greater contrast per unit weight than iodine [31–35]. Another material that has been investigated is tantalum oxide (Ta_2_O_5_, *Z*_Ta_ = 73) nanoparticles, and its use has been evaluated both *in vitro* and *in vivo* [36–39]. Bismuth-based CAs are considered an excellent alternative among metal-containing CAs because bismuth is one of the heaviest and most dense metals (*Z*_Bi_ = 83), with low levels of toxicity. For decades, bismuth has been used in cosmetic and medical formulations, and its toxicity profile has been studied extensively [40–47]. Due to bismuth's high atomic number, materials containing bismuth, such as bismuth sulfide (Bi_2_S_3_) nanoparticles and hybrid nanocrystals of iron oxide and bismuth oxide, have demonstrated excellent *in vitro* and *in vivo* contrast enhancement, long circulation times, and safety profiles [48–53]. Since different metals and nanoparticles have shown to be good X-ray attenuators, the standardization of the quantity of materials needed within cells to produce a detectable signal is difficult to determine. Combinations of different elements, their ratios, and the attenuation capability of each element will significantly influence how much material is needed; thus, independent *in vivo* studies must be conducted for each radiocontrast agent to experimentally evaluate how much material is required per cell to produce a signal in a particular tissue of interest.

Here, we describe the successful internalization of a CNT-based X-ray CA into MSCs. The CA consists of a hybrid material containing ultra-short single-walled carbon nanotubes (20–80 nm, US-tubes) and a Bi(III) oxo-salicylate cluster ([Bi_4_(*μ*-O)_2_(HO-2-C_6_H_4_CO_2_)_8_]·2MeCN) which contains four Bi^3+^ ions in its core (Bi_4_C). This cluster, as with most bismuth compounds, is insoluble in aqueous media which limits its use in biological studies. Carbon nanotubes, although insoluble in water in their pristine state, can be easily modified to increase their suspendability in aqueous media, and CNT-based materials have shown promise as biocompatible platforms for delivering imaging agents into cells in a safe manner [8, 10, 54, 55]. In previous work, we described the use of a CNT-based material containing ∼ 2.6 wt.% of Bi^3+^ (Bi@US-tubes) as an intracellular CA for X-ray CT [7]. Here, we report the performance of a new and considerably improved material (Bi_4_C@US-tubes) as an intracellular contrast agent for MSCs, which contains 20% bismuth by weight.

## 2. Materials and Methods

### 2.1. Preparation of Bi_4_C@US-Tubes

Bi_4_C@US-tubes were prepared as previously reported [56]. Briefly, the US-tubes and the Bi(III) oxo-salicylate cluster were prepared separately and then combined by simply sonicating both together for 1 h in dried tetrahydrofuran (THF). The US-tubes were produced by cutting full-length single-walled carbon nanotubes (SWCNTs) by the fluorination method described elsewhere [57], and the synthesis of the Bi(III) oxo-salicylate cluster ([Bi_4_(*μ*_3_-O)_2_(HO-2-C_6_H_4_CO_2_)_8_]·2MeCN) was performed as previously reported [58] and placed under vacuum for 48 h for solvent removal. For *in vitro* studies, a labeling solution was prepared by suspending Bi_4_C@US-tubes in a 0.17% (*w*/*v*) solution of Pluronic® F-108, a nonionic surfactant, via probe sonication for 5 min. The samples were centrifuged at 3200 rpm for 10 min, and the supernatant was used for the *in vitro* experiments. The bismuth concentration was determined using inductively coupled plasma optical emission spectrometry (ICP-OES, Optima 4300 from PerkinElmer, Inc.) and adjusted to 1 *μ*M.

### 2.2. Stability Challenge of Bi_4_C@US-Tubes

The stability of the Bi_4_C@US-tube material has been previously reported [56]. In the present study, the stability of the Bi_4_C@US-tubes suspended in 0.17% Pluronic® was assessed to determine whether a rigorous sonication process affects the stability of the Bi_4_C@US-tubes when suspended. Aliquots of the labeling solution were placed in centrifuge filter tubes (50 mL tubes, 10 kDa), kept at 37–40°C for 24 h, and finally centrifuged (Figure S1). The supernatant was treated with additions of 70% HNO_3(aq)_ trace-metal grade and 26% HClO_3(aq)_ under heat to digest the organic material. Samples were then analyzed for bismuth by inductively coupled plasma mass spectrometry (ICP-MS, Elan 9000 from PerkinElmer Inc.).

### 2.3. Labeling MSCs with Bi_4_C@US-Tubes

Porcine MSCs isolated from the bone marrow of adult male pigs (three different animals) were intracellularly labeled with Bi_4_C@US-tubes. Prior to labeling, the concentration of the labeling solution was determined by ICP-OES and adjusted to 1.0 mM Bi^3+^. To sterilize the labeling solution, the sample was exposed to UV light for 3 hours with rocking. MSCs were grown in 175 cm^2^ flasks in *α*-minimal essential medium (*α-*MEM) supplemented with 10% fetal bovine serum (FBS) and incubated at 37°C in a humidified atmosphere containing 5% CO_2_ in air. Prior to labeling, cryopreserved cells at the third passage were thawed and then allowed to grow until 70–80% of confluence. The labeling solution was directly added to the culture medium to obtain the desired final concentration. During this process, FBS-free *α-*MEM was used, and FBS was added to the cell cultures 4 h postlabeling to obtain a 10% FBS final solution. Subsequently, cells were incubated and left undisturbed for 20 h. For US-tubes, a 24 h incubation time was found to be optimal to obtain maximum intracellular labeling when incubating MSCs with the material [10]. For this reason, a 24 h labeling protocol was also used here for the Bi_4_C@US-tubes.

For cell collection, MSCs were trypsinized and passed through a 70 *μ*m filter to eliminate large cell aggregates. A density gradient separation, using Histopaque® 1077 (25°C, Sigma-Aldrich), was performed as described elsewhere, [8, 10] to remove free Bi_4_C@US-tubes from the cell suspension. Labeled MSCs were isolated from the interface of the *α-*MEM and Histopaque® layers using a plastic transfer pipette and washed with PBS. Finally, cells were counted using a particle counter (Beckman Counter MultiSizer 3). Experiments with just the Bi_4_C cluster as the labeling agent were not possible to conduct due to the water-insoluble nature of the cluster; thus, unlabeled MSCs were used as control cells. Experiments were performed in triplicate using cells from three different animals unless otherwise specified.

### 2.4. Elemental Analysis of Bi_4_C@US-Tube-Labeled MSCs

Aliquots of cell suspensions were collected in glass scintillation vials to determine the Bi^3+^ ion concentration within the cells by ICP-MS analysis. To prepare the samples for analysis, cells were heated and two alternating aliquots of 500 *μ*L 70% HNO_3(aq)_ trace metal grade and 26% HClO_3(aq)_ were added to digest the organic matter. The samples were allowed to dry between treatments. Finally, the samples were diluted to 5 mL with 2% HNO_3(aq)_ trace metal grade and filtered through a 0.22 *μ*m pore size syringe filter.

### 2.5. Viability of Bi_4_C@US-Tube-Labeled MSCs

Bi_4_C@US-tube-labeled MSCs were prepared as described above. After a 24 h incubation time with the Bi_4_C@US-tubes, labeled cells were collected for the viability study. Positive control (unlabeled MSCs) and negative control (dead unlabeled MSCs, treated with 70% methanol for 20 min) were also studied. To determine the viability of the MSCs, a LIVE/DEAD viability/cytotoxicity assay kit (Invitrogen™) was used. The kit consists of two reagents: calcein AM, which fluoresces green when cells are viable and ethidium homodimer-1 (EthD-1), which fluoresces red when the cellular membrane is compromised. The reagents were added to each sample and cells were incubated in the dark for 20 min at room temperature. Fluorescence-activated cell sorting (FACS) was performed using a LSR flow cytometer (Becton Dickinson).

### 2.6. Subcellular Localization and Label Retention Electron Microscopy

TEM analysis was performed to determine the subcellular localization of the Bi_4_C@US-tubes. Labeled MSCs (300 *μ*M Bi^3+^, for 24 h) and unlabeled MSCs were centrifuged separately at 1500 rpm for 10 min to form a cell pellet (∼1 × 10^6^ cells/pellet). The supernatant was removed without disturbing the cell pellet and fixed with 4% glutaraldehyde. Samples were left undisturbed for 2 days at 4°C. Subsequently, samples were washed with PBS and postfixed with 1% OsO_4_ for 1 h, followed by dehydration with increasing concentrations of ethanol. Finally, samples were infiltrated with acetone and Epon 812 resin and embedded in a mold with 100% Epon 812. Using a Leica EM UC7 ultra-microtome, blocks containing the embedded samples were cut in 1 mm sections and stained with 1% methylene blue and 1% basic fuchsin and also cut in ultra-thin sections (80 nm) and framed on 100-mesh copper grids. Grids were stained with 2% alcoholic uranyl acetate and Reynolds' lead citrate. The grids were examined using a JEOL 1230 TEM instrument equipped with an AMTV 600 digital imaging system. To verify the clearance of the intracellular Bi_4_C@US-tube material from the labeled MSC cultures, the intracellular Bi^3+^ ion content and the amounts found in the culture medium were quantified for up to 72 h postlabeling. MSC cultures from two animals were labeled with Bi_4_C@US-tubes (300 *μ*M Bi^3+^), in triplicate, as described above. After labeling, cells were replated in 6-well plates at 20 × 10^3^ cells/well. Cultures were subjected to medium changes (2 mL) every 24 hours. The 2 mL media collected at 24, 48, and 72 h were centrifuged, and the top 1 mL was removed and filtered with 20 *μ*M membrane. The pelleted material (dead floating cells and cell debris) was resuspended in the remaining 1 mL and used to determine the number of dead cells present in the medium. Elemental analysis was performed by ICP-MS. Bi^3+^ ion concentrations were determined in both the filtered cell culture medium and in the adherent cell monolayer. Dead/detached cells were counted using the Trypan Blue exclusion method.

### 2.7. Population Doubling Time (PDT) Assay

Cell proliferation was measured every 24 hours for up to 144 h using the CyQUANT® NF Cell Proliferation Assay Kit (Invitrogen), which is based on a DNA fluorescent dye which measures the DNA content. MSC cultures previously labeled for 24 hours with Bi_4_C@US-tubes (300 *μ*M Bi^3+^) and unlabeled cells, including cells cultured in regular medium and cells exposed to medium containing 0.17% Pluronic®, were subcultured in 96-well plates (∼1 × 10^3^ cells/well). The CyQUANT reagent was added to triplicate wells after medium removal and incubated for 1 h at 37°C, after which the nuclear fluorescence was measured using a TECAN Safire 2™ microplate reader at 485/528 nm (excitation/emission). To calculate the cell number corresponding to the fluorescence intensities, we used a standard curve previously made by plating a known number of MSCs for all experimental conditions.

### 2.8. Colony-Forming Unit Fibroblast (CFU-F) Assay

The ability of MSCs to self-renew upon Bi_4_C@US-tube labeling was also investigated. Bi_4_C@US-tube-labeled MSCs were plated in triplicate 75 cm^2^ tissue culture flasks at 1500 cells/flask (20 cells/cm^2^). The low plating density allowed cells to grow as individualized colonies. Cells were incubated for 14 days with medium replacement every 3 to 4 days. Flasks were then washed with PBS, fixed with 70% methanol, dried, and stained with Giemsa. Unlabeled MSCs and MSCs treated with 0.17% Pluronic® for 24 h were also plated under the same conditions and used as controls. For the CFU-F enumeration, a stereomicroscope was used.

### 2.9. Three-Lineage Differentiation

To determine whether the Bi_4_C@US-tube material affects the ability of the cells to differentiate into different progenitor cells, unlabeled MSCs and Bi_4_C@US-tube-labeled MSCs were exposed to differentiation-inducing media to promote adipogenic, osteogenic, and chondrogenic differentiation.

For adipogenic and osteogenic differentiation, labeled and unlabeled counterpart MSC cultures were initially plated and grown in 6-well tissue culture plates at ∼20 × 10^3^ cells/well in *α-*MEM. After 24 h, the culture medium in the plates prepared for osteogenic differentiation was replaced with the differentiation medium (*α-*MEM supplemented with 10% FBS, 50 *μ*g/mL ascorbate 2-phosphate, 0.1 *μ*M dexamethasone, and 10 mM *β*-glycerol phosphate). Cultures were maintained in differentiation medium for 14 days with medium changes every 3 to 4 days. To demonstrate osteoblastic differentiation, cultures were stained with Alizarin Red S to detect extracellular calcium deposition.

Adipogenic differentiation medium (*α-*MEM supplemented with 10% FBS, 1% insulin-transferrin-selenium (ITS), 1 *μ*M dexamethasone, 0.5 mM methyl-isobutylxanthine, and 100 *μ*M indomethacin) was added to the cultures when confluence levels reached 50%. Cultures were kept in the differentiation medium for 3 days and subsequently in adipogenic maintenance medium (*α-*MEM supplemented with 10% FBS and 1% ITS) for the following 48 hours. This procedure was repeated twice. To evidence adipogenic differentiation, cultures were stained with Oil Red O and hematoxylin to reveal lipid vacuoles in red and cell nuclei in blue, respectively.

For chondrogenic differentiation, ∼200 × 10^3^ MSCs were transferred into a 15 mL conical tube and centrifuged at 1200 rpm for 5 min, followed by careful removal of the supernatant. Cell pellets were then incubated in chondrogenic differentiation medium (*α-*MEM supplemented with 1% ITS, 40 *μ*g/mL proline, 100 *μ*g/mL sodium pyruvate, 0.1 *μ*M dexamethasone, 50 *μ*g/mL ascorbate 2 phosphate, 10 ng/mL TGF-*β*3, and 0.2 *μ*g/mL BMP6) for 21 days. Differentiation medium was replaced every 3 to 4 days. Upon completion of the assay, cell pellets were fixed with 4% paraformaldehyde, embedded in paraffin, and stained with Alcian blue to reveal accumulation of glycosaminoglycans, an abundant extracellular component of cartilaginous matrices.

### 2.10. X-Ray CT of Bi_4_C@US-Tube-Labeled MSCs

The ability of Bi_4_C@US-tubes to attenuate X-rays once internalized in MSCs was evaluated using a clinical CT scanner (iCT 256 Phillips) at St. Luke's Baylor Hospital, Houston, TX. Bi_4_C@US-tube-labeled MSCs were prepared as described above, followed by centrifugation for 10 min at 1200 rpm to form a cell pellet (∼200 × 10^6^ cells/pellet). Separately, a cell pellet of unlabeled control MSCs was also prepared in a 1.5 mL Eppendorf tube. 500 *μ*L of 5% agar was added carefully on top of the cell pellets and samples were kept refrigerated at 4°C. Data were acquired using the following scanner parameters: tube voltage = 120 kV, pitch = 0.664, gantry rotation time = 0.33 s, mAs/mA = 150/302, and reconstruction slice thickness = 0.625/0.312 cm. For quantitative analysis, three areas per sample were selected as regions of interest (ROI) and measured in Hounsfield units (HU). The reported values are the average measured for axial and coronal views. Analysis was performed using OsiriX v. 4.1.2. 32 bit, an open source software.

### 2.11. Statistical Analysis

All experiments were conducted in triplicate, and values are reported as mean ± standard deviation. All statistical analyses were performed using GraphPad Prism 7.00. The single-factor analysis of variance (ANOVA) test was used to determine statistical significance, and the level of significance alpha was defined at 5%, unless otherwise specified.

## 3. Results and Discussion

### 3.1. Bi_4_C@US-Tube Stability Studies

The preparation of the 1 mM Bi^3+^ labeling solution (Bi_4_C@US-tubes suspended in a 0.17% Pluronic® solution) requires probe sonication, a process that might disrupt the interaction between the Bi_4_C cluster and the US-tubes. To investigate possible detachment of bismuth from the material after sonication, filtration challenge was performed as described above. No detectable bismuth was observed in the filtered solution by ICP-MS. This finding demonstrated that the Bi_4_C cluster remained bound to the US-tubes, even after the vigorous sonication process.

### 3.2. Bi_4_C@US-Tube MSC Labeling, Viability, and Intracellular Retention

**Figure 1:**
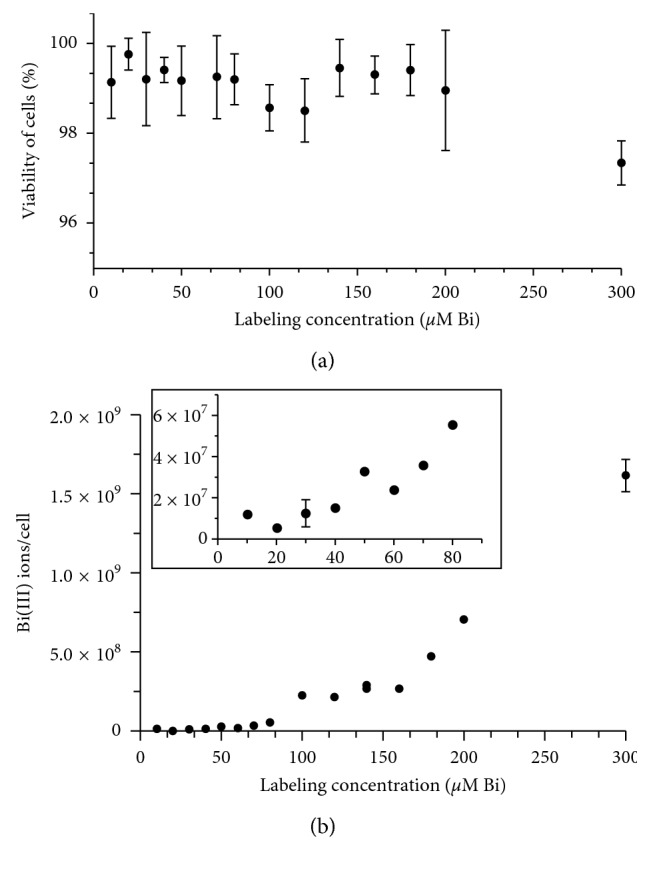
Cell viability and Bi_4_C@US-tube uptake under different labeling concentration. (a) Average cell viability measured by fluorescence activation cell sorting (FACS) analysis. (b) Intracellular incorporation of Bi_4_C@US-tubes inferred by the concentration of Bi^3+^ ion/cell from ICP-MS measurements. The inset shows the plot for the lower concentration range.

MSC cultures isolated from adult male pigs (*N* = 3) were used for the *in vitro* studies. A stock solution (1 mM Bi3+) was added to the culture medium directly. Fifteen different labeling concentrations (10, 20, 30, 40, 50, 60, 70, 80, 100, 120, 140, 160, 180, 200, and 300 *μ*M Bi^3+^) were evaluated. Cell viability under these different concentrations was determined using flow cytometry for cells stained with calcein AM and EthD-1. As seen in Figure 1(a), the viability of the MSCs remained greater than 96% even for the highest concentration studied (300 *μ*M Bi^3+^), demonstrating that MSCs tolerate well the incorporation of Bi4C@US-tubes. Viability for unlabeled MSCs was 97 ± 3% positive for calcein AM. Dead MSCs (methanol induced) were used as a negative control in each experiment and found to be 98 ± 2% positive for EthD-1. More detailed information can be found in Figure S2.

To determine concentrations of intracellular Bi_4_C@US-tubes, MSC cultures were plated and labeled as described earlier. After 24 h incubation, cells were collected, counted, and prepared for elemental analysis of Bi^3+^ ion. The results of these elemental analyses are depicted in Figure 1(b). The uptake of the Bi_4_C@US-tubes varied from 1.2 × 10^7^ to 1.6 × 10^9^ ions of Bi^3+^ per cell. We observed a significant correlation (*p* < 0.0001; *r* ^2^ = 0.86) between intracellular concentrations of Bi^3+^ and the labeling concentration in the medium. The incubations with lower concentrations (10 to 80 *μ*M) produced minimal amounts of bismuth uptake. However, at labeling concentrations higher than 100 *μ*M, greater uptake of the Bi_4_C@US-tube material was observed.

**Figure 2:**
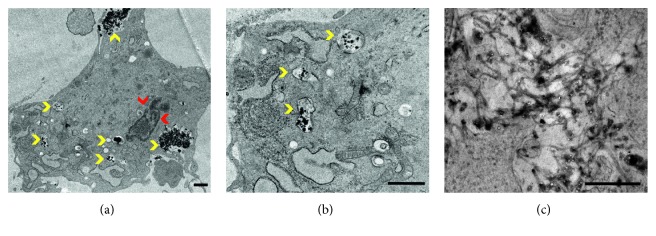
TEM images of Bi_4_C@US-tube-labeled cells. (a and b) Yellow arrows indicate Bi_4_C@US-tubes encapsulated in vacuoles localized in the cytoplasm, while red arrows show the nucleus. (c) An enlarged image of the Bi_4_C@US-tube material, where fiber-like agglomerates can be seen. Scale bar = 1 *μ*m.

TEM was used to further investigate MSC intracellular labeling. To obtain the greatest Bi^3+^-ion loading, 300 *μ*M Bi^3+^ was selected as the labeling concentration, which would also result in the greatest X-ray attenuation. TEM images of the labeled MSCs (Figure 2) showed that Bi_4_C@US-tubes accumulated exclusively in the cytoplasm of the cells, with no evidence of translocation into the nucleus. The same result has been reported for similar materials, such as the Gadonanotubes [10], which are US-tubes loaded with Gd^3+^-ion clusters and other analogous materials [8, 10]. Importantly, the Bi_4_C@US-tubes are the first of a US-tube-based material to become exclusively encapsulated within vesicles in the cytoplasm of the MSCs; however, the encapsulation of US-tube materials within vesicles has been previously observed in cancer cells (Hep3B and HepG2) [59]. In some of the vesicles, the characteristic formation of fiber-like structures that CNT materials often adopt in aqueous media is also clearly visible (Figure 2(c)). The fact that Bi_4_C@US-tubes are seemingly being internalized by the MSC *via* an active transport mechanism might be due to the surface charge of the nanomaterial. Bi_4_C@US-tubes have a slightly more positive charge compared to US-tubes and Gadonanotubes, as confirmed by its zeta potential, obtained using a Malvern Zen 3600 Zetasizer (Table S1). The present TEM data clearly demonstrate that Bi_4_C@US-tubes do not cross the nuclear membrane, but instead, are only enclosed in vesicles which suggests an active cellular transport mechanism, as found for other CNT-based materials used to label MSCs [54].

**Figure 3:**
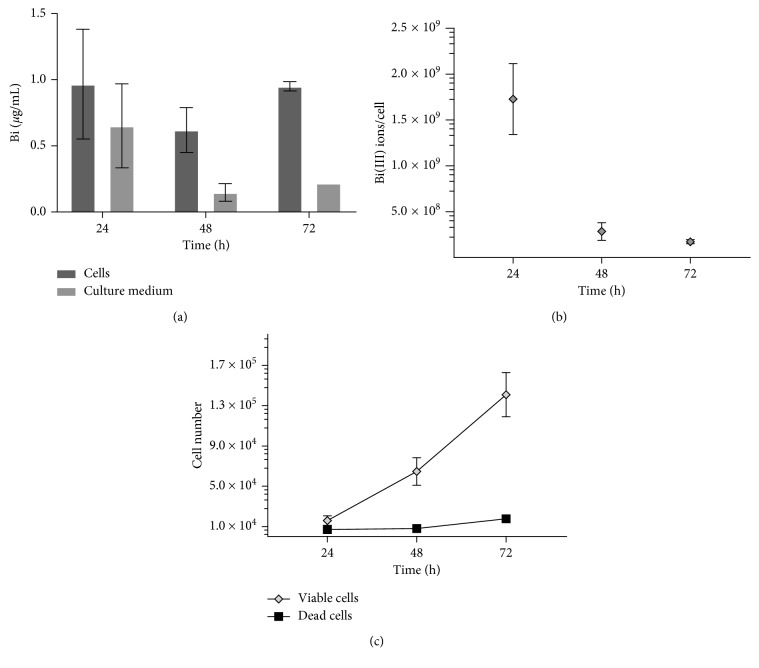
(a) Bi^3+^-ion concentration in cell samples and cell culture medium supernatant over time. (b) Bi^3+^-ion concentration per viable cell over time. (c) Cell numbers for the viable and dead cells after plating. Some error bars are too small to show. Data are presented in mean ± SD.

The likely clearance of incorporated Bi_4_C@US-tube material from cells represents a crucial issue that can impact cell tracking performance. To determine whether intracellular Bi_4_C@US-tubes are released from the cells over time, we investigated the Bi^3+^-ion content in the culture medium and within the cells for up to 72 h as detailed in Section 2.  Figure 3(a) shows the average Bi^3+^ concentration detected in the culture medium, as well as in the cell fraction samples collected at 24, 48, and 72 h after labeling. We observed no significant difference between baseline 24 h Bi^3+^ concentration in the cellular fraction and the two later time points (48 and 72 h) indicating that cells retained incorporated Bi_4_C@US-tubes. The slight decrease in the average of the Bi^3+^ concentration in the cell fraction could be due simply to the initial removal of residual material from the external cellular membrane. The Bi^3+^ concentrations in the filtered culture medium showed significant differences among the three time points, which is a result of the daily medium change.

A detailed representation of the intracellular concentration of the Bi_4_C@US-tube material per viable cell is presented in Figure 3(b). An initial concentration of about 1.7 × 10^9^ ions Bi^3+^/cell was obtained after incubation and replating (24 h), after which a decrease in the metal concentration per cell was observed. This can be explained by the expanding cell numbers that is depicted in Figure 3(c) (attached cells). Also, Figure 3(c) shows the number of dead cells found floating on the medium during the experiment duration. Our results indicate that uptake of Bi_4_C@US-tubes by MSCs is persistent, allows cell growth, and does not affect cell viability.

### 3.3. Proliferative Properties of Bi_4_C@US-Tube Labeled MSCs

**Figure 4:**
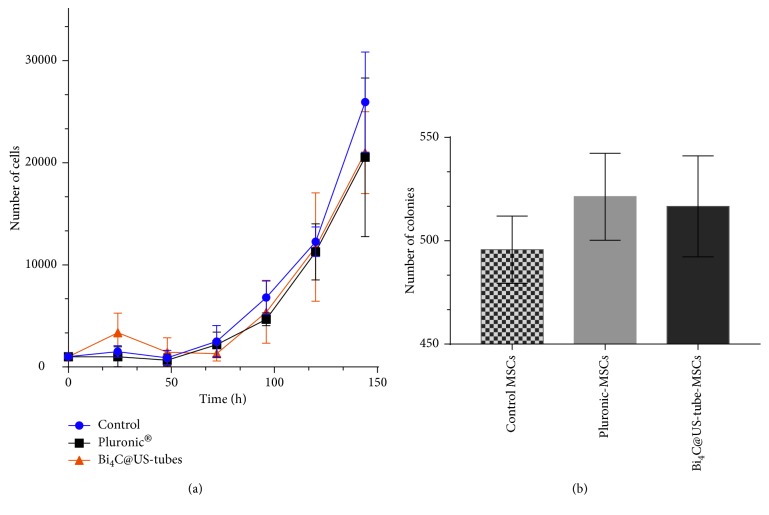
(a) Proliferation and (b) clonogenic assays in control MSCs, MSCs treated with Pluronic®, and Bi_4_C@US-tube-labeled MSCs.

To study the effect of Bi_4_C@US-tubes on MSC proliferative capacity, we determined the population doubling time (PDT) and enumerated colony-forming unit fibroblast (CFU-F) in control (unlabeled and Pluronic®-treated cells) and Bi_4_C@US-tube-labeled MSCs. Growth kinetics is illustrated in Figure 4(a). No difference among the three different conditions was observed. Labeled and unlabeled cells (control and Pluronic®-treated) reached full confluence at 144 h after plating. Also, no difference was found in the PDT values obtained for control cells (19.3 ± 3.1), Pluronic®-treated MSCs (24.8 ± 8.4), and Bi_4_C@US-tube-labeled MSCs (20.1 ± 2.8). Self-renewal, a stem cell hallmark, was evaluated using the CFU-F assay. No statistical difference in CFU-F numbers obtained from plating of Bi_4_C@US-tube-labeled MSCs and those from unlabeled control and Pluronic®-treated MSCs (Figure 4(b)) was observed. These results show that MSC proliferative properties were not altered by Bi_4_C@US-tube incorporation.

### 3.4. Three-Lineage Differentiation

**Figure 5:**
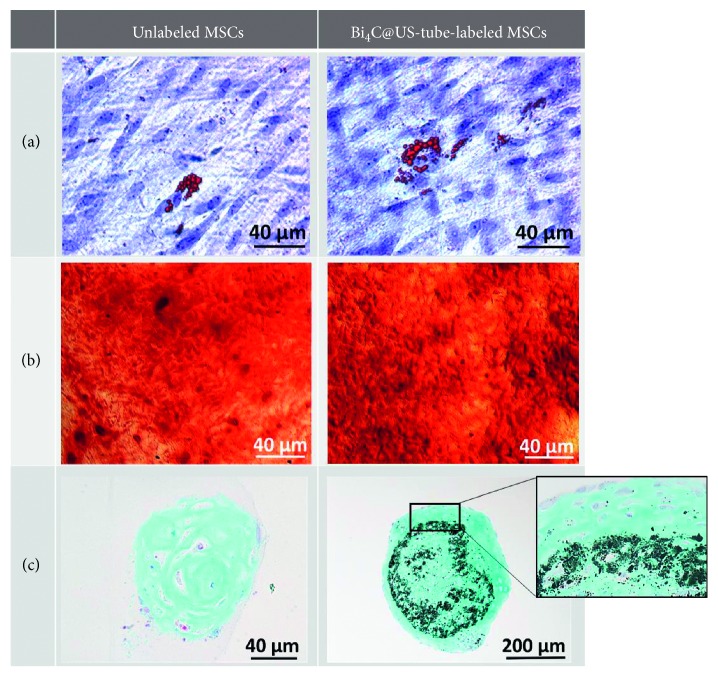
Histochemical staining of unlabeled and Bi_4_C@US-tube-labeled MSCs. (a) Adipocytes are evidenced by the red stain of lipids. (b) Osteoblast activity is demonstrated by extracellular calcium deposits. (c) Hypertrophic chondrocytes are located at the cell pellet periphery.

As multipotent progenitors, mesenchymal cells have the ability to differentiate into fat, bone, and cartilage (adipocytes, osteocytes, and chondrocytes, respectively), upon culture in differentiation-inducing medium. As seen in Figure 5, Bi_4_C@US-tube-labeled MSCs successfully differentiated in fat, bone, and cartilage tissues as evidenced by staining of intracellular lipid vacuoles (in red; A), calcium deposit (in red; B), and glycosaminoglycans (in blue; C). As seen in Figure 5(c), the Bi_4_C@US-tube material remained in the center of the cell sphere, and direct prolonged-exposure (∼3 weeks) to the Bi_4_C@US-tube material did not change the normal behavior of MSCs. This confirms that the labeling of MSCs with the Bi_4_C@US-tube material did not affect MSC differentiation, suggesting that Bi_4_C@US-tube-labeled MSCs may retain their therapeutic potential.

### 3.5. X-Ray CT of Bi_4_C@US-Tube-Labeled MSCs

**Figure 6:**
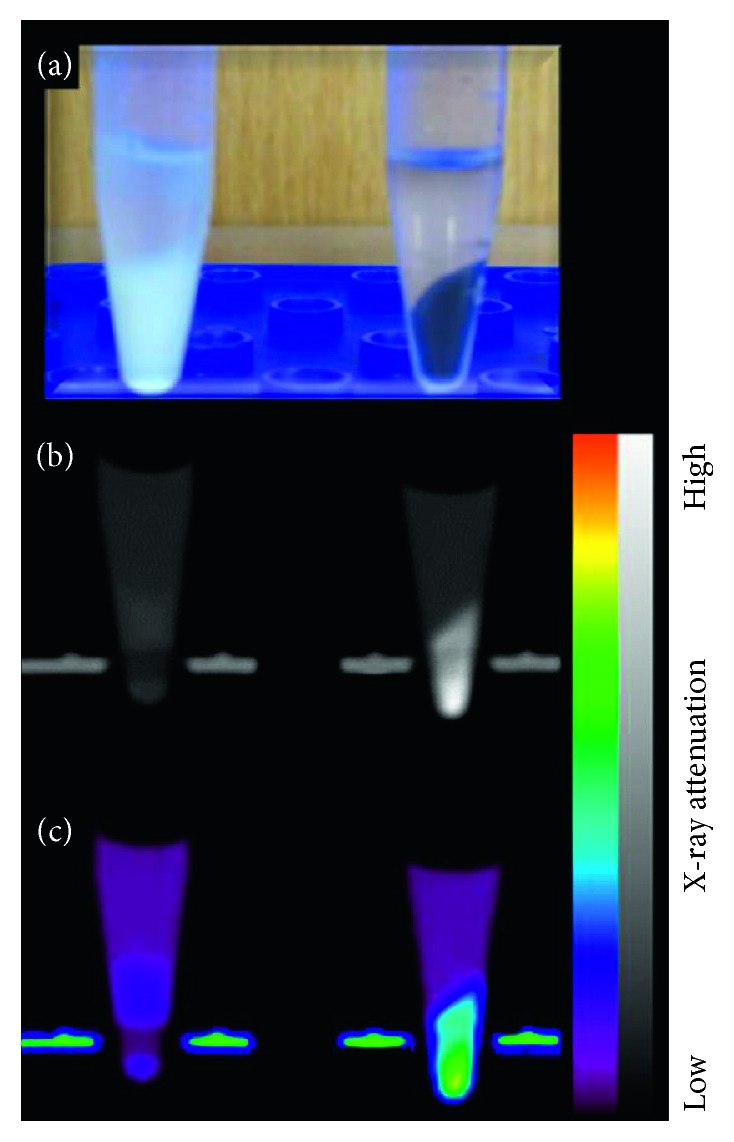
(a) Photograph of the unlabeled MSCs (left) and Bi_4_C@US-tube-labeled MSCs (right). (b) Conventional black and white CT images (coronal view) and (c) color CT images.

The potential of the Bi_4_C@US-tube material to function as an intracellular contrast agent for X-ray CT was evaluated by preparing cell pellets of unlabeled MSCs and Bi_4_C@US-tube-labeled MSCs, as described above.  Figure 6(a) shows Eppendorf tubes containing 200 × 10^6^ cells each. Unlabeled MSCs appear white, while labeled MSCs are dark due to cellular internalization of the Bi_4_C@US-tube material. Analysis of regions of interest revealed HU values, which is an indicator of the ability of the material under study to attenuate X-rays with respect to water (0 HU) and air (−1000 HU). HU values obtained for the control (unlabeled) MSCs and for Bi_4_C@US-tube-labeled MSCs were 30 ± 9 and 214 ± 22, respectively. Thus, the HU value obtained for the Bi_4_C@US-tube-labeled MSCs is approximately 2 times greater than those previously reported for Bi@US-tube-labeled MSCs (110.1 ± 4.9 HU) [7]. The HU values of the different iodinated contrast media used in the clinic in soft tissues range from 100 to 300 HU [60]; hence, the performance of Bi_4_C@US-tube-labeled MSCs is clearly within an acceptable range for X-ray attenuation application of clinical value.

## 4. Conclusion

The work presented here introduces X-ray CT as an alternative imaging technology for the imaging of MSCs. Many other imaging techniques have been investigated for this purpose, such as MRI and optical imaging modalities; however, CT offers some advantages over these imaging technologies such as fast data collection and high spatial resolution. The new CNT-based CA reported here, Bi_4_C@US-tubes, is taken up by MSCs without the use of transfection agents *via* an active transport mechanism to form aggregates within vesicles in the cytoplasm. After internalization of the Bi_4_C@US-tube material within the cells, an increase in X-ray attenuation was obtained, allowing CT images of the labeled MSCs to be brighter compared to unlabeled control cells. The Bi_4_C@US-tubes do not alter the viability, proliferation, or differentiation potential of the MSCs, which suggest that the stem cells retain their characteristic and therapeutic properties. This new intracellular CA material with 20% bismuth by weight is approximately 2 times brighter when localized within MSCs than the first-generation material, Bi@US-tubes, which contained only ∼2.6% bismuth by weight. Taken together, these two works serve as a proof-of-concept example, which demonstrates the feasibility of using an intracellular radiocontrast marker of bismuth to visualize and potentially track stem cells. In the present work, X-ray CT was the imaging modality of choice; however; other X-ray-based technologies, such as conventional X-ray and fluoroscopy could also be used to visualize the labeled cells.

To the best of our knowledge, Bi@US-tubes and Bi_4_C@US-tubes are the first CNT-based materials containing bismuth to be used as intracellular CAs for the imaging of cells by X-ray CT. A similar work has been reported for MSCs labeled with gold nanoparticles and transplanted in a rat model; however, the cell growth after labeling was not evaluated [61]. A similar work with gold nanoparticles was reported for transplanted MSCs for bone regeneration [62]. Other previously reported methods to track cells by CT have not involved the CA being intracellular in nature. For example, alginate-poly-L-lysine-alginate microcapsules containing barium sulfate or bismuth sulfate have been investigated as radiopaque capsules to track human cadaveric islets by encapsulating the cells in the inner space of the capsulates [63]. Similar microcapsules containing gold nanoparticles [60, 64, 65] and perfluorocarbons [66] have also been reported for multimodal imaging, including CT, and for the encapsulation of different types of cells. Therefore, the present Bi_4_C@US-tube formulation (and its Bi@US-tube predecessor) represents significant progress in the development and implementation of intracellular CNT-based CAs containing bismuth for X-ray-based imaging of live cells. Moreover, animal studies still need to be conducted to evaluate the X-ray attenuation capability of the material *in vivo* and its possible eventual elimination from the body, as well as the minimum number of labeled cells needed to produce a detectable signal.

## Data Availability

The data used to support the findings of this study are included within this article and in the supplementary material file. Any other additional information is available upon request.

## Abbreviations

AFM: Atomic force microscopy
Bi_4_C: Bi(III) oxo-salicylate cluster
CA: Contrast agent
CFU-F: Colony-forming unit fibroblast
CNT: Carbon nanotubes
CT: Computed tomography
EDS: Energy dispersive spectroscopy
HU: Hounsfield units
ICP-MS: Inductively coupled plasma mass spectrometry
ICP-OES: Inductively coupled plasma optical emission spectroscopy
MSCs: Mesenchymal stem cells
PDT: Population doubling time
RBM: Radial breathing mode
STEM: Scanning transmission electron microscopy
TEM: Transmission electron microscopy
TGA: Thermogravimetrical analysis
XPS: X-ray photoelectron spectroscopy
US-tubes: Ultra-short carbon nanotubes.

## Supplementary Materials

Supplementary Materials Additional data (raw data obtained by fluorescence-activated cell sorting (FACS) of unlabeled cells, positive control cells, negative control cells, and Bi4US-tube-labeled MSCs and Z-potential values for Pluronic® alone, SWCNTs, US-tubes, Gadonanotubes, and Bi4@US-tubes), as well as a representation of the filtration-challenge process performed in the cell labeling solution, can be found in the Supporting Material file.
