# Supplementary material for: Labeling Stem Cells with a New Hybrid Bismuth/Carbon Nanotube Contrast Agent for X-Ray Imaging
Source: Contrast Media Mol Imaging. 2019 Jun 11;2019:2183051. doi: 10.1155/2019/2183051 (PMC6594287; doi:10.1155/2019/2183051)
Supplement: Supplementary Materials — Additional data (raw data obtained by fluorescence-activated cell sorting (FACS) of unlabeled cells, positive control cells, negative control cells, and Bi4US-tube-labeled MSCs and Z-potential values for Pluronic® alone, SWCNTs, US-tubes, Gadonanotubes, and Bi4@US-tubes), as well as a representation of the filtration-challenge process performed in the cell labeling solution, can be found in the Supporting Material file. [file 2183051.f1.docx]

Supplemental Material

Labeling stem cells with a new hybrid bismuth/carbon nanotube contrast agent for X-ray imaging and tracking

*Mayra Hernández-Rivera^a^, Stephen Y. Cho^a^, Sakineh E. Moghaddam^a^, Benjamin Y. Cheong^b^, Maria da Graça Cabreira-Hansen^b,c^, James T. Willerson^b,c^, Emerson C. Perin^b,c^, and Lon J. Wilson^a^**

^a^Department of Chemistry MS-60, Rice University, P.O. Box 1892, Houston, TX, 77251

^b^CHI St. Luke’s Health - Baylor St. Luke’s Medical Center, 6720 Bertner Ave., MC 2-270 Houston, TX 77030

^c^Texas Heart Institute, 6770 Bertner Ave C350, Houston, TX 77030

*Corresponding author:

Lon J. Wilson

durango@rice.edu

Department of Chemistry MS-60, Rice University, P.O. Box 1892, Houston, TX, 77251


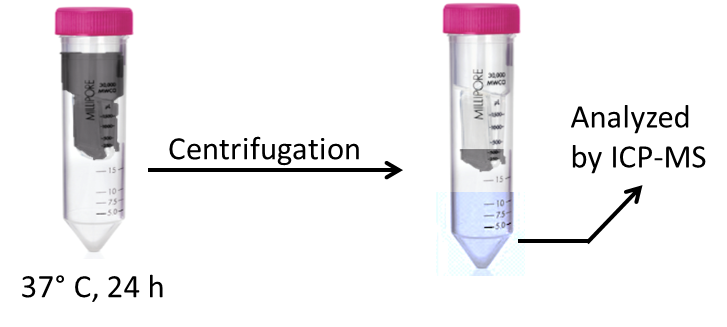


**Figure S1:** Representation of the filtration-challenge process performed in the cell labeling solution (Bi_4_C@US-tubes suspended in Pluronic^®^).


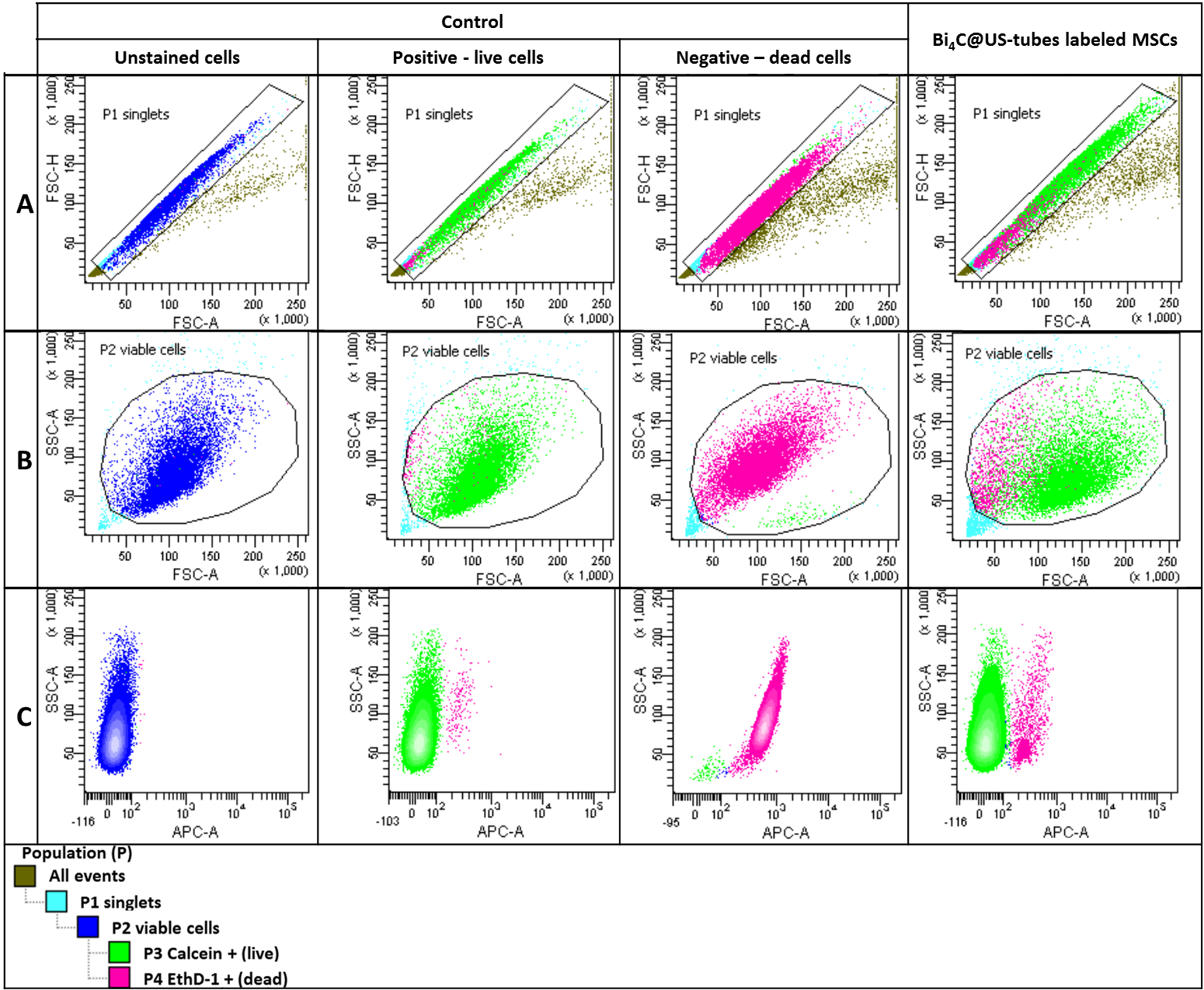


**Figure S2:** Data obtained by fluorescence-activated cell sorting (FACS) of unlabeled cells with not dyes (unstained MSCs), positive control cells (unlabeled live MSCs), negative control cells (unlabeled dead MSCs), and Bi_4_@US-tubes-labeled MSCs (300 µM Bi^3+^). Representation of the gating for the FACS data, meaning, the selection of the regions of interest that determines which population is being analyzed. Since samples contain just one type of cell (MSC), the gating is excluding non-vial cells (debris) or big agglomerates of cells, thus the analyzed cells are viable, single cells. (A) Gating of the single cells, (B) gating for the viable cells, and (C) diagram of the side-scattered light (SSC) and Allophycocyanin (ACP-A) of the viable cells population.

| Suspension | Zeta Potential (mV) |
| --- | --- |
| Pluronic^®^ 0.17% (w/v)* | -19.3 ± 3.4 |
| Full length SWCNTs* | -27.0 ± 1.7 |
| US-tubes* | -53.8 ± 1.8 |
| Gadonanotubes* | -44.3 ± 0.4 |
| Bi_4_C@US-tubes | -21.6 ± 2.3 |

**Table S1:** Z-potential values for Pluronic^®^ alone, SWCNTs, US-tubes, Gadonanotubes and Bi_4_@US-tubes. *Values previously reported in Ref. 1 below.

**Reference**

1. Phounsavath S. RF heating of ultra-short single-walled carbon nanotubes and gadonanotubes for non-invasive cancer hyperthermia. Ph.D. Dissertation, Rice University (2014).
